# Conservation of the mycelia of the medicinal mushroom *Humphreya coffeata* (Berk.) Stey. in sterile distilled water

**DOI:** 10.1016/j.mex.2014.04.002

**Published:** 2014-05-09

**Authors:** Monserrat García-García, Leticia Rocha-Zavaleta, Norma A. Valdez-Cruz, Mauricio A. Trujillo-Roldán

**Affiliations:** Departamento de Biología Molecular y Biotecnología, Instituto de Investigaciones Biomédicas, Universidad Nacional Autónoma de México, AP 70228, CP 04510, México, D.F., México

**Keywords:** Preservation of mycelial cultures of basidiomycetes, Filamentous fungi, Water storage, Mycelium cultures, Castellani's method, Higher basidiomycetes

## Abstract

Currently, there is a growing interest in obtaining and studying the biologically active compounds from higher basidiomycetes, such as *Ganoderma lucidum*, *Lentinus edodes* and *Inonotus obliquus*[1], but the techniques for safe long-term storage are time-consuming, susceptible to contamination, and do not prevent genetic and physiological changes during long-term maintenance [2]. A recent strategy for obtaining biologically active compounds is using mycelia submerged cultures of these mushrooms, cultured under controlled laboratory conditions [1]. However, obtaining spores of these fungi under these conditions is difficult, and in most cases the way to obtain the spores is unknown [1]. Therefore, the strategy for mycelium storage seems to be more appropriated and simple.

• A modification of Castellani's method [3–7] is proposed for higher basidiomycetes, by using the mycelium of *Humphreya coffeata* (Berk.) Stey., whose culture filtrates demonstrated bioactivity against lymphoma cells [8].
• *H. coffeata* (Berk.) Stey. was grown on malt extract agar with filter paper disks that were removed after 4 days, placed in tubes with sterile distilled water, and stored at 4 °C.
• Filter paper disks with *H. coffeata* (Berk.) Stey. stored at 4 °C were confirmed to be viable for up to 18 months, with no visible morphological alterations.

## Graphical abstract

**Figure:**
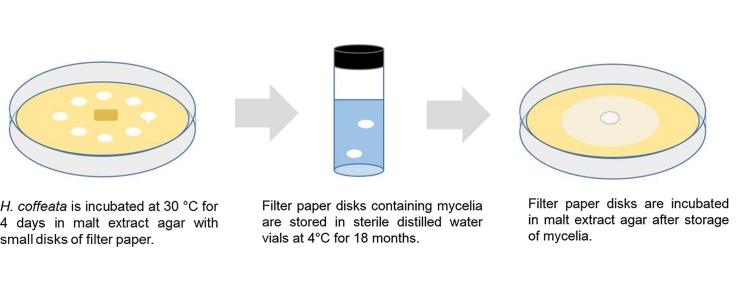

## Method details

There are various methods of conservation, some of the most commonly used are repeated subculturing, lyophilization (unsuitable for most basidiomycetes) and cryopreservation [2,9–11]. However, some of these methods are not compatible with all fungi due to the particular characteristics of each species [1]. We used the method described by Castellani [3–5] with some modifications for the conservation of the mycelia of the higher basidiomycete *Humphreya coffeata* (Berk.) Stey., since it has been reported that this method ensures the viability of isolates for 1–20 years depending on the species [5,6,12,13]; however, it has not been used for higher basidiomycetes.

### Preparation of the malt extract agar, FZM agar and vials

Petri dishes measuring 110 mm × 25 mm were filled with 30 mL of malt extract agar (MEA) or FZM agar; filter paper disks of 5 mm diameter (Whatman^®^ No. 4) were manually prepared and sterilized, and then placed on the filled Petri dishes with the mycelia. MEA contains (in g/L) malt extract 20.0, peptone 1.0, dextrose 20.0, and agar 20.0. [14]. FZM agar [8] contains (in g/L) glucose 35.0, yeast extract 2.5, peptone 5.0, KH_2_PO_4_·H_2_O 1.0, MgSO_4_·7H_2_O 0.5, thiamine 0.05, and bacteriological agar 18.0, pH 5.5 [15]. Then, 10 mL sterile glass vials were pre-sterilized for 21 min at 121 °C (TOMY ES-315 autoclave), and 4.0 mL of distilled sterile water was added to each vial.

### Inoculation of mycelia in malt extract agar

**Fig. 1:**
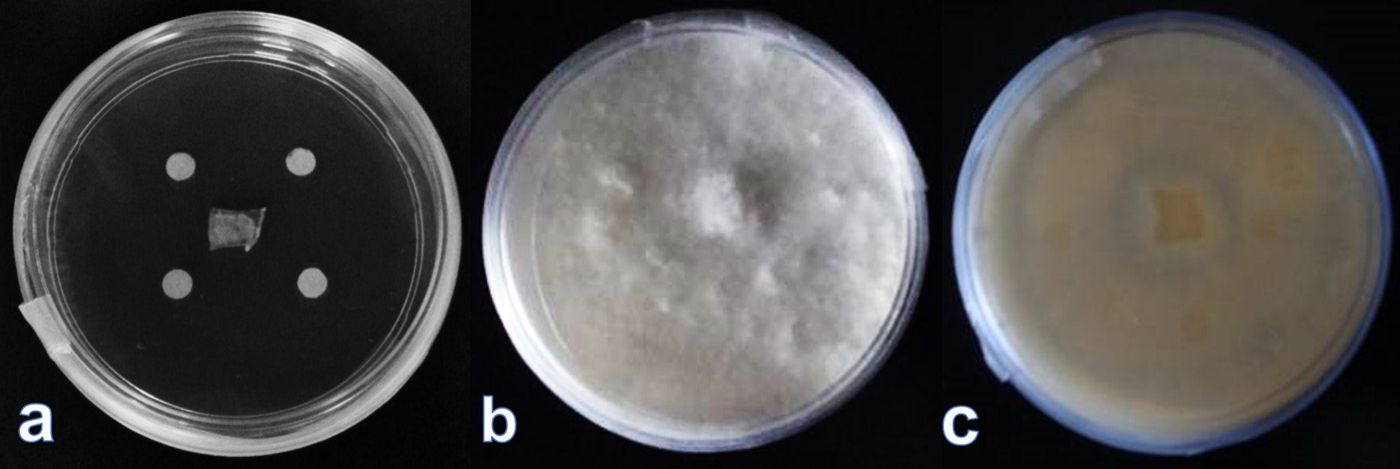
(a) Petri dish with malt extract agar inoculated with *Humphreya coffeata* (Berk.) Stey., showing how the filter paper disks are placed. (b) Cultivation of *H. coffeata* (Berk.) Stey. growing in the Petri dish. (c) The bottom of the Petri dish with filter paper disks covered by mycelium.

The central area of the Petri dishes with MEA or FZM agar media was inoculated with *H. coffeata* (Berk.) Stey. (Fig. 1a), and the 5-mm filter paper disks (Whatman^®^ No.4) were placed around the inoculum. Thereby, on growing the mycelium of *H. coffeata* would cover the Petri dish, including the filter paper disks, as seen in Fig. 1b. The Petri dishes were incubated at 30 °C for 4 days (FELISA FE-293A, México).

### Storage in sterile distilled water

**Fig. 2:**
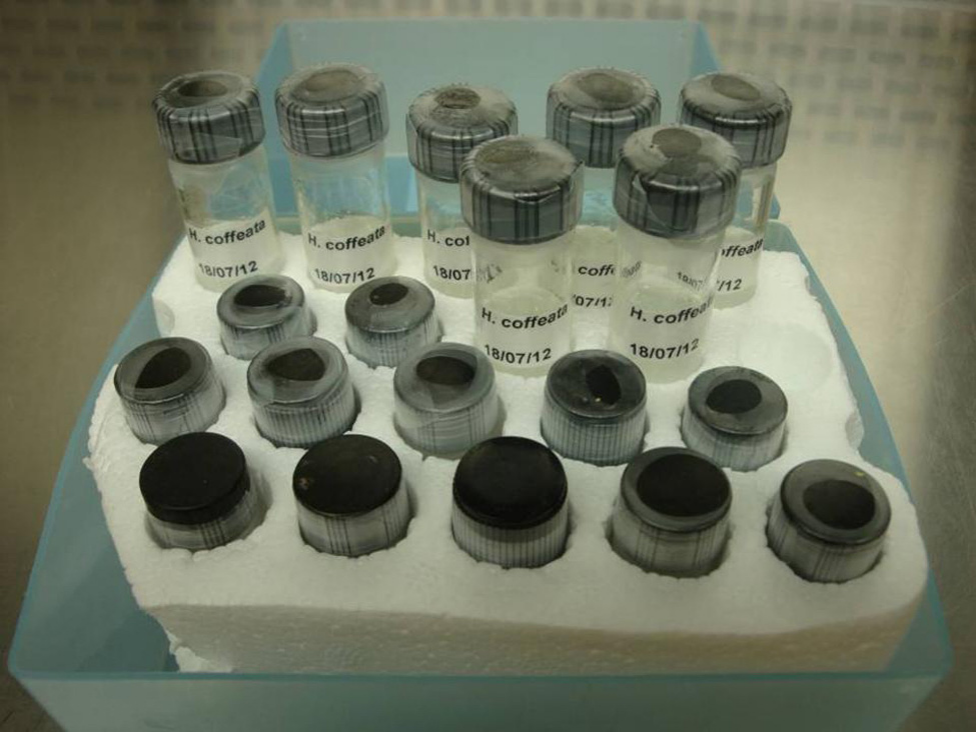
Vials ready to store, containing sterile distilled water and the filter paper disks carrying mycelium of *H. coffeata* (Berk.) Stey. that were removed from the Petri dish under sterile conditions.

After a 4-day period the mycelial growth covered the Petri dish. Then the filter paper disks were carefully removed under sterile conditions, and placed into vials containing 4.0 mL of sterile distilled water. A minimum of 50 vials were prepared. All vials were closed and sealed with 2 cm Parafilm^®^ M (Sigma–Aldrich, USA) strips. Finally, all vials were stored at 4 °C (Fig. 2).

### Viability of the basidiomycetes in sterile distilled water

**Fig. 3:**
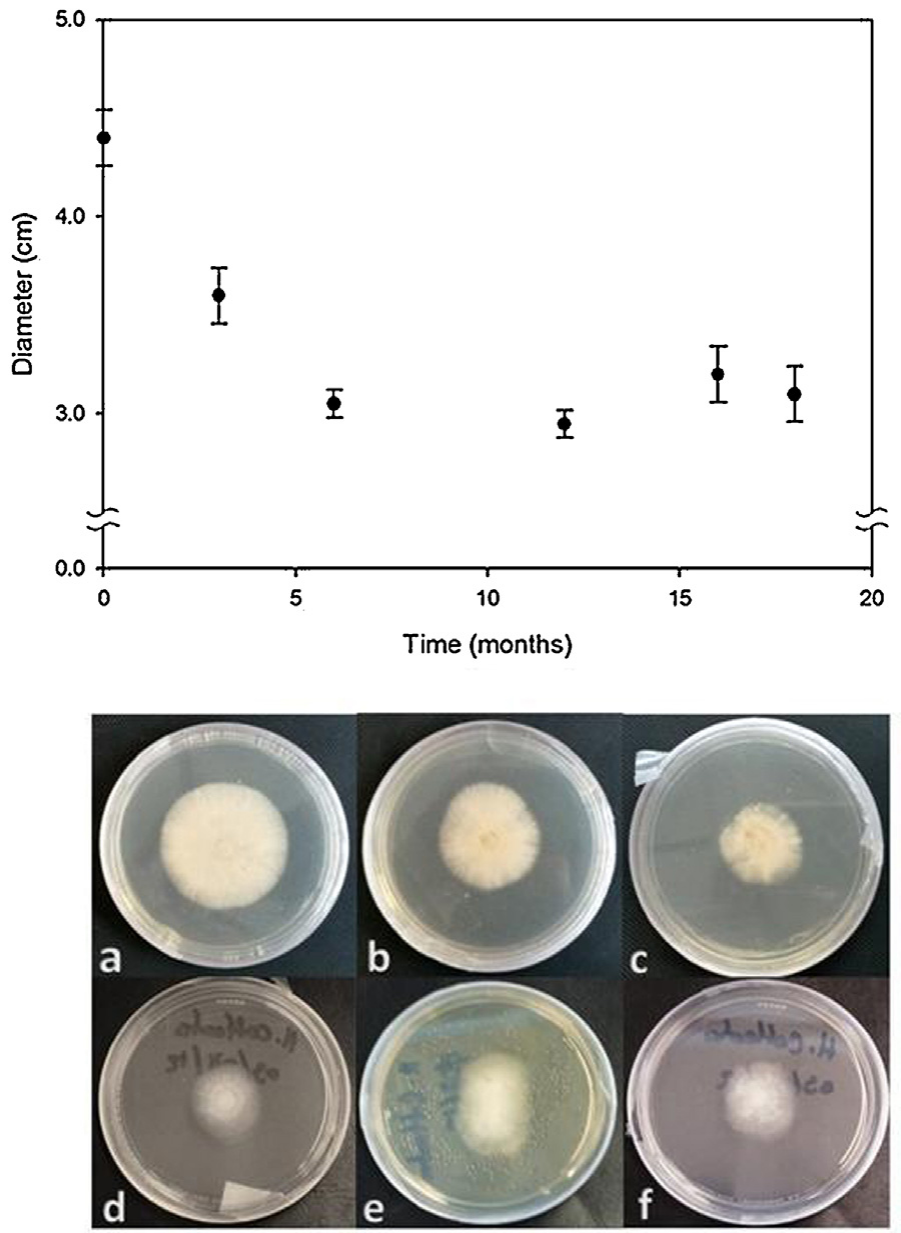
Shelf life test. Filter paper disks stored in sterile distilled water for 0 (a), 3 (b), 6 (c), 12 (d), 15 (e), and 18 (f) months were inoculated in Petri dishes containing FZM agar medium and incubated at 30 °C for 4 days. Upper panel – plot showing the growth (diameter) of *H. coffeata* (Berk.) Stey. mycelium after 4 days of incubation. Lower panel – representative photograph of *H. coffeata* (Berk.) Stey. mycelium growing after preservation in distilled water for the indicated months. All viability trials were made at least in triplicate.

The shelf life of *H. coffeata* (Berk.) Stey. attached to filter paper disks (Whatman^®^ No. 4) and stored in sterile distilled water at 4 °C was evaluated at 0, 3, 6, 12, 15 and 18 months. All viability trials were made at least in triplicate. The culture medium for the evaluation was FZM agar. Growth was assessed macroscopically (Fig. 3), viability was determined by measuring the growth diameter formed by the mycelial culture in FZM agar, and the culture was incubated at 30 °C (FELISA FE-293A, México) for 4 days [4,8].

The conservation of mycelium in filter paper disks of *H. coffeata* in sterile distilled water assured a high viability of cultivation for 18 months (Fig. 3). There were no visible morphological changes, or contamination by bacteria or other fungi. This suggests that this method, in addition to being easy and economical, is suitable for the conservation of higher basidiomycetes such as *H. coffeata*. It should be taken into consideration that the time of viability to ensure this method depends on the species of fungus to store [3–7].
